# Supplementary material for: Are carbon emissions trading and green financial instruments synergistic? -Comprehensive quantitative research based on content analysis
Source: PLoS One. 2024 Mar 7;19(3):e0298601. doi: 10.1371/journal.pone.0298601 (PMC10919601; doi:10.1371/journal.pone.0298601)
Supplement: S1 Data — (ZIP) [file pone.0298601.s001.zip › Data/地级市Z_F工作报告.zip!/╣π╢1⁄2/╜╥╤⌠╩╨/└┤╫╘╣1⁄2╓┌║┼í╛┬φ┐╦╩2╛▌═°í┐_2011.docx]

各位代表：

现在，我代表市人民政府向大会报告政府工作，请予审议，并请政协各位委员和其他列席人员提出意见。

一、“十一五”时期工作回顾

　　“十一五”时期是我市改革开放和现代化建设取得辉煌成就的五年，也是建市以来经济增长最快、发展质量最好、综合实力显著增强、人民群众得到更多实惠的五年。五年来，在省委省政府和市委的正确领导下，在市人大及其常委会和市政协的监督支持下，市政府坚持以邓小平理论和“三个代表”重要思想为指导，深入贯彻落实科学发展观，全面实施“一三二”发展思路，化危机迎挑战，抓机遇促发展，励精图治，奋力赶超，圆满完成了2010年和“十一五”各项目标任务。

　　——经济发展实现重大跨越。主要经济指标五年全部实现翻一番以上，2010年生产总值首次突破千亿元大关，达到1008.99亿元，比上年（下同）增长19.6%，五年年均增长16.9%；人均生产总值17364元，增长18.6%，年均增长15.9%；地方财政一般预算收入38.65亿元，增长33.9%，年均增长28.1%，其中税收收入29.04亿元，增长31.6%，年均增长28.2%.投资、消费、出口协调拉动经济快速增长，2010年完成全社会固定资产投资564.07亿元，增长43.3%，五年年均增长35.8%；社会消费品零售总额446.62亿元，增长31.2%，年均增长25.3%；进出口总额36.3亿美元，增长21.6%，年均增长21.2%，其中外贸出口30.8亿美元，增长22%，年均增长22.2%.招商引资取得重大突破，五年共引进项目1563个，计划投资总额4322.32亿元，中石油广东石化炼油项目前期工作顺利完成；中海油粤东LNG一体化项目、中电投揭阳物流中心项目已经奠基；惠来电厂1、2号机组建成投产，3、4号机组及电网送出工程基本建成。商贸流通繁荣活跃，建成揭阳（国际）金属材料市场、普宁国际服装城、阳美玉都广场、宝德数码广场等一批大型专业市场，连续成功举办5届揭阳国际玉器节、4届揭阳国际五金不锈钢制品博览会、3届普宁国际衬衣节和2届揭西生态旅游文化节。大力开拓国内外市场，分别在郑州、合肥、武汉等地成功举办揭阳产品展销会，先后组团赴泰国、阿联酋（迪拜）、美国（拉斯维加斯）等国家开展外贸展销活动，为保增长、保民生、保稳定作出了重要的贡献。

　　——产业竞争实力显著增强。自主创新能力明显提升，五金机械、纺织服装产业被列为省市“共建支柱产业”，揭东经济开发区被列为国家新型工业化（装备制造）示范基地，建成国家不锈钢制品质量监督检验中心，五年新认定中国高新技术企业43家；新增省级民营科技企业55家；列入省现代产业500强企业16家、自主创新100强企业3家、战略性新兴产业骨干（培育）企业4家；新增国家、省级企业技术中心13个，省级工程技术研发中心13个，省级技术创新专业镇12个；荣获国家专利优秀奖1项，新增专利授权2834项；累计注册商标33012件，新增中国驰名商标11件、省著名商标70件，中国名牌产品2个、省名牌产品8个。产业和劳动力“双转移”有效推进，珠海（揭阳）产业转移工业园被列为省级示范园，大南海国际石化综合工业园获省批准设立。五年累计完成工业投资615.36亿元、企业技术改造投资102.67亿元，新增规模以上工业企业1917家、上市公司2家、投资500万元以上工业项目1315个；2010年规模以上工业增加值438.66亿元，增长38.9%，五年年均增长34.5%.新增限额以上商业企业（含个体户）3602家，国家4A级旅游景区1个、3A级旅游景区3个。三次产业结构由2005年的15.8：48.8：35.4调整为2010年的10.9：57.4：31.7，工业化进程明显加快。

　　——城乡面貌发生巨大变化。海陆空立体交通网络基本形成，揭阳潮汕机场、厦深铁路揭阳段已分别完成总投资的74%和55.5%；神泉港区获批成为国家对外开放一类口岸，惠来电厂10万吨级煤码头建成交付使用；五年累计改造国、省道241.5公里，新（改）建农村公路2628公里，全市公路通车总里程已达6359公里，建成汕揭高速公路揭阳段，开工建设潮州至惠州高速公路揭阳段。完成《揭阳市城镇体系规划》、《揭阳市城市总体规划》、《揭阳市土地利用总体规划》等13个重大规划编制（修编）工作。深化城市投融资体制改革，扎实推进市区建设两个“30亿工程”，先后改造建设临江北路、临江南路、黄岐山大道、新阳路、莲花大道等一批市政道路工程，建成榕江大型音乐喷泉、揭阳楼及广场、念慈体育馆等一批重点市政配套设施，成功创建省“双拥”模范城和省卫生城市，深入开展国家卫生城市和省文明城市创建活动，城市形象得到大幅提升；各县（市、区）五年累计投入资金78.26亿元，建设城区市政项目584个，城镇发展环境得到明显改善。2010年全市城镇化率49.26%.农业农村经济稳步发展，建成农业标准化示范区47个、省级现代农业园区7个、市级以上农业龙头企业27家、农民专业合作组织152个； 2010年农林牧渔业总产值169.02亿元，增长5.1%，五年年均增长4.6%；累计投入水利建设资金31.07亿元，完成230座病险水库除险加固任务；乡镇通村道路全部实现硬底化，乡镇和行政村通班车率分别达到100%、92.34%，行政村卫生所实现全覆盖，建成村文化室799个，农村面貌发生喜人变化。

　　——社会建设取得丰硕成果。顺利完成省下达人口控制目标，2010年人口出生率11.24‰，人口自然增长率6.13‰，符合政策生育率96.22%，榕城区、揭东县进入“国优”行列，普宁市、惠来县由三类地区升为二类地区。节能减排取得明显进步，“榕江环保一路行”深入开展，水环境综合整治工作扎实推进，市区和4个县城污水处理厂建成投产。加强国土资源管理，完成“三旧”改造235个项目面积1万亩，开发补充耕地6万亩。海域使用管理得到加强。林业生态建设取得新突破，完成集体林权制度主体改革任务，森林覆盖率提高至53.1%.安全生产主要指标得到有效控制，没有发生产品质量、食品药品重大安全事故，市场经济秩序进一步规范。全面完成“五五”普法任务。综治信访维稳工作取得新成效， 2010年每万人刑事发案数比全省平均值低79.6%，社会治安持续稳定好转，连续三次获得“全省综治工作优秀市”称号。

　　——人民生活得到明显改善。大力推进基本公共服务均等化，五年累计投入教育资金135.1亿元，新（扩）建中小学902所，增配教师2.04万名，2010年高中毛入学率达到84.9%（比2005年提高39.8个百分点），高考累计录取人数10.7万人，中小学教师工资福利待遇“两相当”基本落实，代课教师问题得到妥善解决；市卫生学校新校区、市人民医院新住院大楼建成交付使用，68%的基层医疗机构实施国家基本药物制度；成功举办广东省第三届粤东侨博会、广州亚运会火炬传递揭阳站活动、市第五届运动会和第一、二届特色文化节，新增国家级非物质文化遗产项目9个、省级非物质文化遗产项目29个，潮剧《还官记》喜获“文华优秀剧目奖”。 2010年城镇居民人均可支配收入14907元，增长13.2%，五年年均增长10.2%；农民人均纯收入6128元，增长12.8%，年均增长8.2%；农村劳动力累计参加职业技能培训20.09万人、转移就业55.35万人，新增城镇就业9.5万人。2010年金融机构各项存款余额967.03亿元，增长18.48%，五年年均增长21%；各项贷款余额407.06亿元，增长21.45%，年均增长27.4%.累计新增机动车12.47万辆。建设保障性住房面积8.98万平方米，解决城镇住房困难家庭1736户。全市职工基本医疗保险、居民基本医疗保险覆盖率均达89.9%，新型农村合作医疗参合率99.2%，13.57万名城乡居民享受最低生活保障。残疾人社会保障和服务体系不断完善。实施扶贫开发“双到”帮扶项目1165个，投入资金2.93亿元，2.14万户贫困户实现了脱贫目标。累计解决农村饮水不安全人口131.52万人。圆满完成对口支援汶川卧龙镇恢复重建任务。

　　——政府自身建设步伐加快。坚持定期向人大及其常委会报告工作、向政协通报情况，密切与各民主党派、人民团体的联系。五年累计承办省、市人大代表议案、建议241件，政协委员提案、建议意见516件，全部得到办理和答复。开通“手机信访”，推进网络问政，拓宽群众反映问题、表达诉求渠道。规范行政决策程序，建立政府常务会议学法制度，实行行政首长问责制，依法行政工作得到不断加强。顺利完成市政府机构改革任务。全面取消市级审批事项，实施12项机关效能制度，对重点企业实施“绿卡”保护制度，制订出台影响机关效能和执行力行为责任追究办法等相关规定，建成市、县、镇、村四级实时电子监察网络，机关服务效能不断提升。审计监督得到新加强。广播电视和新闻出版事业跃上新台阶。物价、统计、供销、气象、地震、人民防空、国防动员、民族宗教、外事侨务、对台事务、文史档案等工作不断进步。与泰国南邦市缔结为友好城市。

　　各位代表，过去五年，我们走过了不平凡的历程。五年的实践让我们更加深刻地认识到，贯彻落实好科学发展观，必须把加快发展作为第一要务，坚定不移调结构，脚踏实地促转型，确保经济持续、快速、协调发展；必须走特色发展道路，充分发挥自身优势，抢占发展高地，着力打造特色经济、特色城市、特色文化；必须以大项目带动大发展，锲而不舍抓招商，攻坚克难求突破，努力推动赶超进位、后来居上；必须坚持以人为本，完善社会管理，扩大公共服务，促进社会公平正义，提高人民群众幸福感；必须弘扬求真务实、真抓实干的良好作风，强化执行力建设，创新抓落实体制机制，高效办事，干净干事。这些宝贵经验是五年来积累的重要精神财富，也是今后政府工作必须继续坚持的重要原则。

　　过去五年，成绩来之不易。这是省委省政府和市委正确领导的结果，是各级人大及其常委会和政协监督支持的结果，是全市人民共同奋斗、社会各界大力支持的结果。在此，我谨代表市人民政府，向全市各条战线的干部群众，向驻揭部队、武警官兵、人民警察和各民主党派、人民团体、各界人士，向一直以来关心支持我市改革发展的港澳台同胞、海外侨胞、外出乡亲以及国际友人，表示衷心的感谢并致以崇高的敬意！

　　在看到成绩的同时，我们也清醒地认识到，我市经济社会发展仍存在不少困难和问题，主要是：经济总量不大，运行效益不佳；产业结构调整不快，自主创新能力不强；社会事业发展仍较滞后，节能减排和安全生产压力较大，社会管理和改善民生任务较重；机关作风建设有待加强，等等。我们对此要高度重视，采取有效措施，认真加以解决。

二、“十二五”时期奋斗目标和主要任务

　　“十二五”时期是我市加快转变经济发展方式、推动跨越发展的攻坚时期，也是可以大有作为的重要战略机遇期。根据省委省政府的总体部署和市委关于“十二五”规划的建议，市政府组织编制了《揭阳市国民经济和社会发展第十二个五年规划纲要（草案）》，提请本次大会审议。《规划纲要（草案）》在总结“十一五”规划实施情况的基础上，分析了“十二五”宏观环境和面临的挑战，提出了今后五年经济社会发展的目标任务和工作措施。 “十二五”时期我市经济社会发展总的指导思想是：坚持以邓小平理论和“三个代表”重要思想为指导，深入贯彻落实科学发展观，紧紧围绕科学发展这一主题、加快转变经济发展方式这一主线、“加快转型升级、建设幸福广东”这一核心，深入实施“一三二”发展思路，树立大思想，发展大经济，建设大城市，打造大文化，构筑大保障，同心同德，忠诚奉献，力拔粤东“五年大变化”头筹，争拔粤东“十年大发展”头筹，为实现一代而强、振兴揭阳打下坚实基础。

　　“十二五”时期经济社会发展的主要目标是：生产总值年均增长23.8%，地方财政一般预算收入年均增长34.7%，经济发展方式转变取得明显进展，社会事业蓬勃发展，民生福祉显著改善，改革开放全面深化，发展环境日臻完善。

　　实现“十二五”规划目标，必须坚持跨越发展、赶超进位，坚持协调发展、统筹兼顾，坚持创新发展、内外并举，坚持和谐发展、民生优先，坚持可持续发展、走文明发展道路，努力实现更好质量、更快速度、更高水平的发展。重点要在“五个着力”上狠下功夫：

（一）着力发展特色经济，建设富强揭阳。狠抓结构调整，加快转型升级，积极构建以重化产业和现代服务业为“双核心”、以传统优势产业为重要支撑、以高新技术产业和战略性新兴产业为重要引领的现代产业体系。深入实施大项目带动战略和“双转移”发展战略，构筑沿江、沿海、中部、西部“两纵两横”四大产业带，进一步优化产业发展布局。坚持内生增长与外向带动并抓、扩大投资与促进消费并举，强化招商引资，突出资本运作，活跃商贸流通，拓展内外需求，迅速做大经济总量。力争市区商业基地面积达到11000亩，普宁达到5000亩，建成立足广东、辐射全国、面向世界的现代服务基地。大力推进自主创新，全面开展质量强市活动，不断提高产业竞争实力，推动经济发展向创新驱动转变。

（二）着力打造宜居城乡，建设魅力揭阳。以改善人居环境为目标，以提高城镇化水平为重点，以“三旧”改造为抓手，加快打造彰显揭阳独特魅力的岭南水城和特色城市群。理顺市区管理体制，拓展城市发展空间，完善市政配套功能，深入开展国家卫生城市、省文明城市等创建活动，不断提升城市形象品位，努力把市区建成人口超120万的宜业宜居大城市。狠抓交通、水利、环保等基础设施建设，强化节能减排和生态保护，改善城乡生产生活环境。统筹城乡区域协调发展，加大扶贫开发力度，提高农业产业化发展水平，建设社会主义新农村。

（三）着力提升文化软实力，建设文明揭阳。加快文化强市建设。大力弘扬忠诚奉献精神，打造“自强自信、创业创新、和谐开放、感恩奉献”特色文化，为揭阳大发展提供强大精神动力。实施科教兴市战略，全面提高各类教育发展水平，加快普及学前至高中阶段15年教育。大力实施文化惠民工程，完善公共文化服务体系，建设一批重点文化设施，打造一批文化艺术精品，促进城乡公共文化服务均衡发展。深化文化体制改革，繁荣发展文化事业，做大做强特色文化产业。

（四）着力保障和改善民生，建设和谐揭阳。坚持以人为本，统筹经济社会发展，扎实推进以改善民生为重点的社会建设，让人民群众享有更多优质的公共服务，不断增强幸福感和自豪感。深化医药卫生体制改革，加强重大疾病防控，保障人民群众身体健康。完善就业服务体系，积极开发就业岗位，扩大就业规模，提高城乡人民收入水平。坚持广覆盖、保基本、多层次、可持续的方针，加快建立覆盖城乡居民的社会保障体系。加强社会管理，创新体制机制，巩固发展和谐稳定的社会局面。

　　（五）着力转变政府职能，建设法治揭阳。深化行政管理体制改革，优化组织结构，完善行政运行机制，加快实现政企、政资、政事分开。大力发展基层民主，畅通社情民意反映渠道，完善科学民主决策机制，以良好的政风促行风带民风。加强机关效能建设，创新政府管理方式，深化政务公开，强化行政监督和行政问责，全面推进依法行政，建设“为民、务实、清廉、高效”政府。

三、2011年工作安排

　　2011年是实施“十二五”规划的开局之年，也是揭阳力拔粤东“五年大变化”头筹的决胜之年。政府工作的总体要求是：深入贯彻落实科学发展观，全面贯彻十七届五中全会、省委十届七次和八次全会、粤东地区工作会议、粤东地区（揭阳）现场会和市委四届八次全会精神，紧紧围绕“加快转型升级、建设幸福广东”这个核心，全面实施“一三二”发展思路，更加突出产业结构调整，更加突出自主创新，更加突出重点项目建设，更加突出社会事业发展，更加突出保障和改善民生，全力推动赶超进位、后来居上，力拔粤东“五年大变化”头筹，为“十二五”开好局、起好步。

　　经济社会发展的主要预期目标是：生产总值增长24%左右；单位生产总值能耗下降3.43%，二氧化硫、化学需氧量、氨氮、氮氧化物和单位生产总值二氧化碳排放量有所下降；规模以上工业增加值增长31%；全社会固定资产投资增长45%；社会消费品零售总额增长28%；外贸出口增长18%；地方财政一般预算收入增长31.5 %；居民消费价格涨幅控制在4%左右；城镇登记失业率控制在3.5%以内；人口自然增长率控制在6.7‰以内；城镇居民人均可支配收入增长13.2%，农民人均纯收入增长13%。实现上述目标，必须突出抓好以下八方面工作：

（一） 加快产业转型升级，推动经济更好更快发展

提升产业核心竞争力。扎实推进自主创新，鼓励和支持企业开展技术改造，设立工程技术和产品研发机构，与高校科研院所建立产学研合作关系，参与国家、行业和地方标准制订工作，创建品牌商标，开展自主营销，推动企业由制造加工向研发设计升级，由贴牌生产向品牌营销转型。推进质量强市建设，积极构建公共技术服务平台，加快科技创新成果转化，不断提高产业发展水平和竞争力。争取今年新增中国高新技术企业6家，省级民营科技企业12家，省级工程技术研发中心5个，省级企业技术中心5个，确保国家不锈钢制品质量监督检验中心顺利通过验收并投入使用，抓好国家机织制品质量监督检验中心申报工作。

调整优化工业结构。推动工业向适度重型化方向发展，重点发展电力能源、石油化工、大型装备制造等重化产业，完善产业链条和服务配套，加快形成新的产业集群。瞄准高端装备制造、太阳能光伏、生物制药、新材料、半导体照明等战略性新兴产业，组织实施一批重点新兴产业技术攻关及产业化示范项目，加快形成具有较强竞争力的新兴产业体系。全面落实促进中小企业发展各项政策措施，加强省市共建支柱产业建设，继续抓好“三个新增100”，着力打造一批产值超50亿元、100亿元的大型集团和骨干企业，发展壮大传统优势产业。

大力发展现代服务业。适应办大工业的新需要，加快发展港口物流、金融保险、信息咨询、电子商务等生产性服务业，优化提升现代商贸、旅游休闲、医疗保健等消费性服务业，逐步提高服务业对经济增长的贡献率。加快10大国际商贸城建设，实施“万村千乡”市场工程，抓好“农超对接”和平价商场建设，办好玉器节、衬衣节、五金不锈钢制品博览会等地方特色节会，搞活搞旺城乡消费市场。加强区域经济协作交流，继续推进揭货“北上西进”，认真筹办揭阳产品（重庆）展销会，进一步扩大揭阳品牌影响力。

促进外贸持续较快发展。鼓励企业创建出口品牌，提高传统出口商品附加值和科技含量，增强产品国际竞争力。大力开拓东盟、中东、南美等新兴市场，深度开发北美、西欧等高端市场，组团参加荷兰国际品牌采购展，确保外贸出口平稳较快增长。建立完善进口贸易促进平台，鼓励企业扩大重要原材料和先进适用技术进口，推动进出口均衡发展。完善口岸基础设施，抓好揭阳潮汕机场口岸对外开放申报工作，争取扩大揭阳港对外开放水域，不断优化贸易投资环境。

　　（二）狠抓重点项目建设，夯实跨越发展基础

全力推进基础设施建设。加快潮州至惠州高速公路揭阳段建设，争取开工建设揭阳至惠来、汕头至湛江高速公路揭阳段。确保揭阳潮汕机场、厦深铁路揭阳段按期建成投入运营。改造建设一批国省道和农村公路项目。抓好榕江航道浚深工程。加快实施引韩城乡供水工程，逐步解决市区及揭东东部资源性缺水问题。抓紧推进城乡水利防灾减灾和病险水库除险加固工程，加快建设市三洲拦河闸应急重建工程后续项目；动工建设惠来赤岑水闸、邦山水闸，加紧实施惠来西水东调工程、普宁白坑湖水库综合整治工程。大力推进城乡电网改造建设，满足用电需求和送电需要。

抓好重点项目落地建设。优化投资环境，加强跟踪协调，完善立项、用地等相关手续，争取引进的项目尽快落地建设。全力推动重大项目建设取得新突破，力争上半年中石油广东石化炼油项目核准并开工建设，中海油粤东LNG一体化、中电投揭阳物流中心、靖海蓝海通用码头等项目核准并进入实质性施工，中广核揭阳核电、揭阳疏港铁路列入国家规划，惠来电厂3、4号机组并网发电。

加大招商引资工作力度。健全招商引资工作机制，推动工作重心、主体、责任“三下移”，办好6月份全市招商引资大会和9月份各县（市、区）招商引资验收现场会，确保全年招商引资规模超过1500亿元。深入实施“乡贤回归”工程，争取更多外出企业家回乡投资置业。大力引进大型国有企业、国内龙头民营企业、世界500强企业到揭阳落户。积极承接发达地区产业转移，切实提高珠海（揭阳）产业转移工业园办园水平。加快大南海国际石化综合工业园、空港经济区、英歌山工业区规划建设，打造新的经济增长极。

　　（三）加强农业基础建设，提高“三农”工作水平

稳定和扩大粮食生产。全面落实粮食和农业生产各项扶持政策，广泛调动农民种粮积极性。认真贯彻落实中央一号文件精神，加强农田水利建设，提高农业综合生产能力。深入挖掘耕地潜力，加大土地开发整理力度，稳定粮食播种面积。加快现代标准农田建设，力争整治改造中低产田6万亩。加强农科推广应用和服务，确保农业稳产高产。

大力发展现代农业。调整农业生产结构，提高农业产业化水平，加快转变农业发展方式。强化农业龙头企业和农民专业合作社的带动作用，大力发展地方特色农副产品，提高精深加工水平，促进农业增效、农民增收。培育发展油茶、珍贵树种等特色林业。加强标准渔港建设，提高现代渔业发展水平。

加快扶贫开发步伐。全面推进扶贫开发“双到”工作，积极开展产业扶贫和智力扶贫，全力加快帮扶项目建设，如期实现脱贫目标。加大资金投入，大力推进“雨污分流”处理和农村垃圾集中处理工程。抓紧解决农村群众饮水安全问题。

加强新农村建设。扎实推进农村综合改革，继续抓好农村土地承包管理工作，全面完成集体林权制度配套改革。深入推广玉湖经验，做好村民委员会和社区居民委员会换届选举工作。加快农村基础设施建设，完善农村公共服务体系，改善农村生产生活条件。

（四）强化经济管理服务，提高运行质量和效益

加强经济运行调节。强化价格监测预警，加大价格调节基金征收力度，抓好粮油等重要物资调度和储备，保持价格总水平基本稳定。全面落实“米袋子”、“菜篮子”行政首长负责制，确保主要农副产品有效供给。完善补贴制度，保障困难群众基本生活。调节供需关系，促进房地产市场健康发展。加大知识产权保护力度，严厉打击制假售假、商业欺诈等违法行为，营造公平竞争、健康有序的市场环境。

深化经济领域改革。完善国有资产管理体制，妥善处置历史遗留问题。创新投资合作机制，发挥政府投融资平台作用，确保重点建设项目对资金的需求。加强对民间投资的服务、管理和引导，鼓励社会力量投资基础产业和基础设施，捐资建设公共服务设施、兴办社会事业。抓好水价、电价综合改革，促进城乡公用事业健康发展。

提高财税征管水平。综合运用财税政策手段，涵养和培植财源。加大经济户口清理力度，深入挖掘增收潜力，创新税费征管手段，按序时进度均衡组织财税收入。深化财政各项改革，提高科学化精细化管理水平。优化财政支出结构，保障民生和重点支出的需要。坚决落实厉行节约各项要求，严格控制一般性支出，切实提高财政资金使用效益。

强化金融信贷服务。加强社会信用体系建设，密切银企合作，扩大信贷规模，提高企业直接融资比重。争取揭西农村信用合作联社改制为农村商业银行，成立普宁村镇银行，新增小额贷款公司6至8家、融资担保机构4至6家。积极推动优质企业上市，力争今年新增上市公司3家。

（五）抓好规划建设管理，力促城镇化水平有新提升

拓展城市发展空间。全面组织实施《揭阳市城镇体系规划》、《揭阳市城市总体规划》，抓紧编制市区控制性详细规划、修建性详细规划和各项专项规划，打破行政区划限制，优化和延伸城市发展空间。积极推进“三旧”改造，依法完善历史用地手续，促进建设用地“二次开发”。 加快渔湖新区开发建设步伐。

完善市政配套功能。继续抓好市区建设两个“30亿工程”，抓紧推进榕江两河四岸景观配套建设和内城河整治，积极完善市政道路网、绿道网、地下管网体系，确保按期完成水利市政工程BT项目、市文化中心9大项目建设任务，加快建设市区观音广场，启动顺风大桥、太极广场、紫峰山公园等建设项目，进一步改善城市公共服务设施。加强城市资源经营管理，继续落实各县（市、区）城区建设筹资任务，增强城镇辐射带动功能，加快城乡一体化进程。力争今年全市市政建设投入达到100亿元，城镇化率达到61%.提升城市形象品位。加强城市网格化管理，加大动态执法巡查工作力度，切实提高城市行政综合执法水平。深入开展市容市貌、环境卫生、道路交通等集中整治行动，着力解决城市 “脏、乱、差”等突出问题。加强市民公共道德教育，规范城市公共秩序，提升城市文明程度。争取今年市区获得“广东省创建文明城市工作先进城市”称号，两个县城进入“广东省创建文明县城工作先进县城”行列。

（六）加强和创新社会管理，促进社会和谐稳定

建设生态文明。强化节能减排，抓好4个中心镇污水处理厂和8个垃圾无害化处理场建设，确保今年城镇污水处理率达到60%，城市生活垃圾无害化处理率达到90%.认真落实土地管理共同责任制度，严肃查处和整治违法违规用地行为。完善殡葬设施，巩固殡改成果。加强海洋生态保护，提高城乡绿化水平，构建生态安全屏障。

创新社会管理。加强人口计生服务管理，抓好综合改革创建活动，积极探索推进人口计生工作科学发展新路子。健全社会工作体系，加强社区管理和服务，充分发挥社会组织在构建和谐社会中的积极作用。全面落实党政领导干部安全生产“一岗双责”制度，坚决防范和遏制重特大事故发生。健全公共卫生、食品药品安全预防预警体系，切实提高应急管理工作水平。

推进平安揭阳建设。完善综治信访维稳体系，加大社会矛盾纠纷排查调处工作力度，把不稳定因素化解在基层和萌芽状态。健全社会治安防控体系，加强城乡社区警务、人民调解、群防群治等基层基础建设。深入开展社会治安十大专项整治行动，着力解决重点地区治安突出问题，依法严厉打击各种刑事犯罪活动，营造和谐稳定社会环境。

（七）加大民生保障力度，增强人民群众幸福感

优先发展教育事业。全面实施《揭阳市教育事业发展规划（2011-2020年）》，继续落实教育资金筹措、校舍建设和提高师生素质三大任务，推动各类教育健康、协调、可持续发展。编制实施学前教育三年行动计划，切实解决儿童“入园难、入园贵”问题。调整优化中小学布局，促进义务教育均衡发展。加快普及高中阶段教育，大力发展职业教育、技工教育。支持特殊教育和民办教育发展。办好揭阳职业技术学院和潮汕职业技术学院，加快筹办华南化工学院。加强师德师风建设，提高教学水平。

推进文化强市建设。深入开展忠诚教育和感恩奉献教育，传承地方特色文化，打造鲜明的城市文化形象。加快市区重点文化项目建设，完善县、镇公共文化设施，新（改）建一批文化馆、图书馆、博物馆以及村级文化室（广场），积极推进广播电视村村通、农村电影放映、农家书屋建设、文化信息资源共享等文化惠民工程，切实提高城乡公共文化服务水平。加快发展平面传媒、广播影视、文化旅游等产业，培育发展新型文化业态。加强重点文物、非物质文化遗产申报、保护和开发利用。

提高全民健康水平。加强医疗卫生服务体系建设，配套完善市级和县级医院、乡镇卫生院、社区卫生服务中心、村卫生所，促进公共卫生服务均等化。全面推广落实惠来县深化医药卫生体制改革试点经验，加快实施国家基本药物制度，扎实推进公立医院改革，加强镇、村医疗人员业务培训，切实解决群众反映强烈的“看病难、看病贵”问题。推进城乡医疗保障一体化，狠抓医保扩面征缴，增强医保基金筹资能力和抗风险能力。广泛开展全民健身运动，增强城乡居民身体素质。

加强劳动就业和社会保障。实施积极就业政策，稳定和扩大就业。做好高校毕业生、农村转移劳动力、城镇就业困难人员就业工作，加强职业技能培训，提高劳动者就业能力。完善城镇职工养老保险市级统筹制度，逐步扩大新型农村社会养老保险制度试点范围。健全住房保障体系，积极推进公租房建设，抓紧解决城镇低收入家庭住房困难问题。落实城乡低保制度，加强社会福利机构建设，大力发展民间慈善事业，提高救灾救助工作水平。

大力发展其他事业。巩固省“双拥”模范城创建成果，做好国防动员、人民防空工作。提高统计服务质量。发展老龄、妇幼和残疾人事业，做好民族宗教、外事侨务、对台事务、气象地震、档案史志等工作。

在这里，我代表市人民政府承诺，今年将集中力量重点办好10项民生实事：一是提高城乡低保补助水平， 二是基本完成三年扶贫开发任务，三是稳定和扩大就业，四是提高基层医疗卫生服务水平，五是促进基础教育全面发展，六是完善社会保障体系，七是大力发展民生水利事业，八是加强城乡文化设施建设，九是抓好保障性安居工程建设，十是大力改善城乡交通环境。

（八）推进民主法制建设，树立政府良好形象

坚持民主施政。自觉接受人大、政协监督，认真办理人大代表和政协委员建议提案。广泛听取各民主党派、工商联、无党派人士和各人民团体的意见建议，积极发挥经济顾问、专家咨询组作用，建立完善重大事项集体决策、举行听证、社会公示等制度，切实提高科学、民主、依法决策水平。加快网络问政平台建设，加大政务公开力度，主动接受社会监督和舆论监督，推动公众有序参与政务管理。

坚持依法行政。贯彻落实国务院、省政府关于推进依法行政工作的决策部署，全面实施法治揭阳建设五年规划，启动“六五”普法教育，建立健全依法行政配套制度，不断提高各级政府依法行政能力和水平。加强行政复议工作，强化行政执法监督。抓好榕城区大部门体制改革试点工作，扎实推进富县强镇和简政强镇事权改革，理顺权责关系，优化职能结构。

坚持高效理政。深化行政审批制度改革，规范行政审批和行政许可事项，完善行政服务体系，推进行政审批权相对集中改革，建设行政服务中心，大力推行“并联审批”、“网上审批”、“网上办事”和“一站式”服务，切实提高行政效能。整合政务网络资源，建立统一电子政务平台，完善电子监察系统。对重点工作继续实行插“红旗”制度，强化督查督办和绩效考核，提高行政执行力和公信力。

坚持从严治政。全面落实《廉政准则》“52个不准”，扎实推进政府系统党风廉政建设和反腐败斗争。完善行政问责制和责任追究制，强化政府层级监督和行政监察、审计监督，严肃查处违法违纪案件，坚决纠正损害群众利益的不正之风，树立清正廉洁的政府形象。

　　各位代表！美好蓝图已经绘就，幸福航程又将开启。让我们在省委省政府和市委的坚强领导下，万众一心、锐意进取，开拓创新、扎实工作，为建设富民强市、和谐文明新揭阳而努力奋斗，以优异成绩迎接中国共产党成立90周年！
